# 

**Published:** 1905-09

**Authors:** 


					﻿SUBSCRIPTION $1.00
ATLANTA PUBLISHED MONTHLY.
JOURNAL-RECORD
SURfi f ON Gt N E R A L'SO^ J J MEDICINE.
Successor to Atlanta dical and Surgical Journal, Established 1855,
and Southerr fledical Record, Established
Ga., Septej^ BFifc 1905.	A No. 6.
				

